# Histopathological growth pattern of liver metastases as an independent marker of metastatic behavior in different primary cancers

**DOI:** 10.3389/fonc.2023.1260880

**Published:** 2023-10-27

**Authors:** Ali Bohlok, François Richard, Valerio Lucidi, Antoine El Asmar, Pieter Demetter, Ligia Craciun, Denis Larsimont, Alain Hendlisz, Jean Luc Van Laethem, Luc Dirix, Christine Desmedt, Peter Vermeulen, Vincent Donckier

**Affiliations:** ^1^ Surgical Oncology, Institut Jules Bordet, Université Libre de Bruxelles, Brussels, Belgium; ^2^ Laboratory for Translational Breast Cancer Research, Department of Oncology, KU Leuven, Leuven, Belgium; ^3^ Abdominal Surgery, Hôpital Erasme, Université Libre de Bruxelles, Brussels, Belgium; ^4^ Pathology, Institut Jules Bordet, Université Libre de Bruxelles, Brussels, Belgium; ^5^ Digestive Oncology, Institut Jules Bordet, Université Libre de Bruxelles, Brussels, Belgium; ^6^ Hepatogastroenterology, Hôpital Erasme, Université Libre de Bruxelles, Brussels, Belgium; ^7^ Translational Cancer Research Unit, Gasthuiszusters Antwerp Hospitals and University of Antwerp, Antwerp, Belgium

**Keywords:** liver, metastases, histopathological growth pattern, prognostic, marker

## Abstract

Surgical resection can lead to prolonged survival in patients with isolated liver metastases (LM) from various primary cancers. However, there are currently no validated predictive markers to discriminate between these oligo/argometastatic patients, who will benefit from surgery, and those with diffuse metastatic behavior in whom surgery will be futile. To evaluate whether the tumor microenvironment, or histopathological growth pattern (HGP), of LM reflects the type of metastatic progression independently of the origin of the primary cancer, we analyzed a combined series of patients who underwent surgery for colorectal LM (N=263) or non-colorectal LM (N=66). HGPs of LM were scored in each patient to distinguish between desmoplastic HGP (all LM showing a complete encapsulated pattern) and non-desmoplastic HGP (at least one LM with some infiltrating-replacement component). In the entire series, 5-year overall and progression-free survival were, 44.5% and 15.5%, respectively, with no significant differences between colorectal and non-colorectal LM. In patients with desmoplastic HGP, 5-year overall and progression-free survival were 57% and 32%, respectively, as compared to 41% and 12%, respectively, in patients with non-desmoplastic-HGP (p=0.03 and 0.005). Irrespective of cancer origin and compared to traditional risk factors, desmoplastic HGP was the most significant predictor for better post-operative overall survival (adjusted HR: 0.62; 95% CI: [0.49-0.97]; p=0.035) and progression-free survival (adjusted HR: 0.61; 95% CI: [0.42-0.87], p=0.006). This suggests that the HGP of LM may represent an accurate marker that reflects the mode of metastatic behavior, independently of primary cancer type.

## Introduction

Patients with liver metastases (LM) from different primary cancers represent a very heterogeneous population in terms of tumor biology, sensitivity to systemic treatments, and indications for surgery. Despite this heterogeneity, long-term survival and occasional cures can be observed in patients who undergo surgical resection of isolated LM of different origins. This is well known in patients with colorectal liver-only metastases, in whom surgery represents the first therapeutic option to consider, allowing long-term survival and potential cure in 25%-40% of the cases (1, 2). This is also the case in patients with LM from diverse primary cancers of digestive and non-digestive origins, frequently regrouped as non-colorectal LM. In these cases, even if their indications remain ill-defined, local approaches, including surgical resection, are now more frequently used, aiming to enhance overall tumor control but also potentially leading to prolonged progression-free survival (3–9). This suggests that, beyond the variability of primary tumors and selection processes, a fraction of these patients with isolated LM could share similar characteristics of restricted metastatic progression, so-called “oligo”- or “argo-metastasis” (10–12). Currently, however, there is no reliable marker to distinguish beforehand the patients who will benefit from surgery from those in whom a limited number of LM corresponds to the first manifestation of diffuse aggressive metastatic disease, in whom surgery will be ineffective. To illustrate this, there is still a significant proportion of patients who undergo curative-intent resection of colorectal or non-colorectal LM that rapidly recur after surgery, and carry a very poor postoperative prognosis (3, 13–17). Accordingly, the identification of (bio)-markers for different metastatic behaviors represents a major objective to improve the individualization of onco-surgical management of these patients.

Distinct histopathological growth patterns (HGPs) have now been identified in LM from colorectal and various non-colorectal origins (18–25). Two main HGPs have been established in consensus guidelines (26): the desmoplastic HGP (dHGP), in which a fibrous rim surrounds the metastasis, and which is associated with angiogenesis and numerous immune cells at the tumor-to-liver interface (TLI), and the infiltrating or replacement HGP (rHGP), in which cancer cells grow directly into the liver parenchyma, replacing the hepatocytes and thus coopting liver sinusoidal blood vessels, with minimal or absent immune infiltrate at the TLI. At the present, both in patients who undergo surgery for colorectal or non-colorectal LM, better postoperative survival has been reproducibly reported in patients with dHGP LM as compared to patients with non-dHGP LM (21, 24–26). This suggests that, beyond major differences in primary tumor biology, LM form different cancers could present similar histopathological features resulting from the interactions between cancer cells and liver microenvironment. On this basis, we hypothesized that categorization of the tumor microenvironment of LM according to their HGP may accurately reflect the overall mode of metastatic progression, irrespective of primary tumor histology.

To verify this hypothesis, i.e., to evaluate whether HGP of LM could represent a cancer-agnostic biomarker, we analyzed the prognostic value of HGP in a combined series of patients who underwent surgical resection of LM from various origins, adjusted for other potential prognostic factors.

## Methods

**Figure 1:**
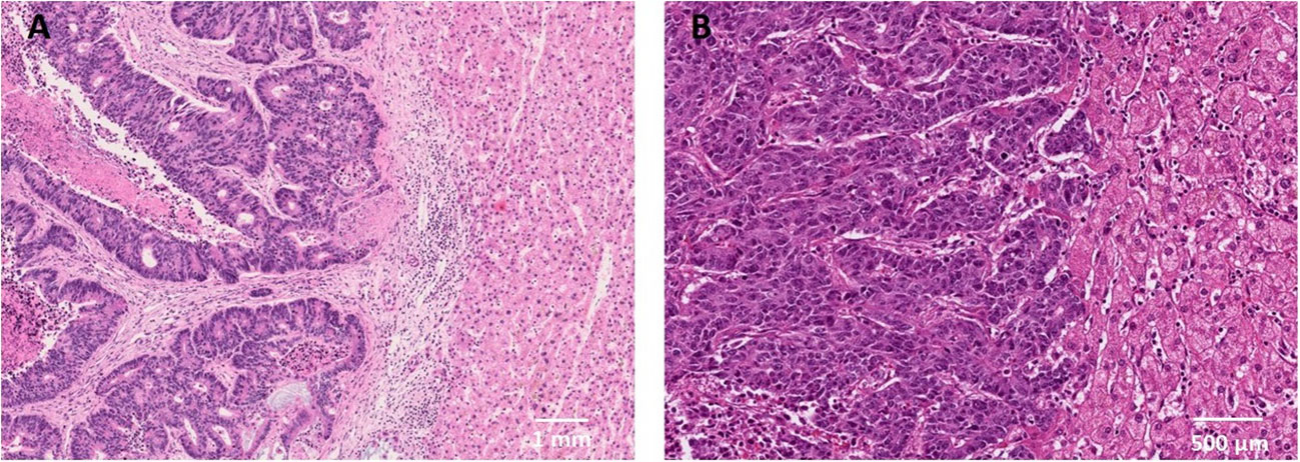
Histopathological growth patterns in liver metastases and their association with survival. **(A)** LM with a dHGP (H&E staining) showing the desmoplastic rim that separates the tumor tissue from the liver parenchyma. **(B)** LM with a rHGP (H&E staining) showing regions where cancer cells grow into the liver cell plate and replace the hepatocytes. Cancer cells are in contact with the hepatocytes. H&E, hematoxylin and eosin staining.

A series of patients who underwent surgery for LM of colorectal and non-colorectal origin between January 2005 and December 2017 at the university hospitals of the Université Libre de Bruxelles was reviewed (Ethical Committees: CE2953 and P2019/232). In each patient, all available hematoxylin/eosin-stained sections of resected LM were analyzed and scored for HGP according to consensus guidelines (26). Patients with incomplete surgical resection, exclusive local destructive treatment with radiofrequency or microwave ablation, complete pathological response to preoperative treatment, or poor tissue conservation were excluded. Clinicopathologic data were evaluated, including primary tumor characteristics, neoadjuvant and adjuvant treatment for primary tumors, LM characteristics, and preoperative and postoperative treatment for LM. HGPs were scored by experienced pathologists (PDM and PV), blinded to clinical data, describing 3 patterns (26): the dHGP (**Figure 1A**), the rHGP (**Figure 1B**), and the rare pushing HGP (detailed in **Supplementary Material 1**). The dHGP was defined as the presence of a characteristic fibrotic rim surrounding the tumor with no direct liver cell-to-cancer cell interaction. In this pattern, the blood supply relies on angiogenesis, demonstrated by endothelial proliferation and regions of high vessel density. In this form, numerous inflammatory/immune cells are present at the TLI. The rHGP was defined as the absence of a peri-tumor fibrotic rim. In these cases, cancer cells replace the hepatocytes, mimic the architecture of liver parenchyma, and coopt the liver sinusoidal vasculature for blood supply. In this form, there is often minimal or absent inflammatory infiltrate at the TLI. As the presence of any non-dHGP has been established as the most discriminant factor for poor prognosis in colorectal LM (26), LM were classified as dHGP when this pattern represented 100% of the TLI of all analyzed LMs and as non-dHGP in the other cases. Progression-free survival (PFS) and overall survival (OS) were evaluated as endpoints for the survival analyses and calculated as the time from resection of LM to recurrence or death. Survival estimates were obtained using the Kaplan-Meier method and compared with the log rank test. Neoadjuvant and/or adjuvant chemotherapy for primary tumor, preoperative and/or postoperative chemotherapy for LM surgery (yes vs no), nodal status of the primary tumor (positive vs negative), number of metastases (multiple vs single), synchronicity (yes vs no), and the type of LM (colorectal vs non-colorectal) were considered as adjustment variables. P-values were 2-sided and considered significant when p<.05. Detailed methods can be found in the **Supplementary Methods**.

## Results

Data for a total of 329 patients were reviewed, including 263 patients with colorectal LM and 66 with non-colorectal LM (detailed in **Supplementary Table 1**). As compared to patients with colorectal LM, patients with non-colorectal LM were younger, had fewer locally-advanced primary tumors, had longer progression-free intervals between primary tumor and surgery for LM, less frequently had multiple LM, and more frequently received systemic treatment after liver surgery (**Table 1**). Overall, 18% of the whole population had dHGP LM, 18.6% in the colorectal LM group and 13.6% in the non-colorectal LM group (Fisher’s test: p=0.47). No association was observed between the HGP and clinicopathologic variables, except for an enrichment of non-dHGP in patients with breast cancer LM (p=0.01), (detailed in **Supplementary Table 2**).

**Table 1:** Characteristics of the population.

Characteristics	ALL LM (n=329)	CRLM (n=263; 80%)	NCRLM (n=66; 20%)	p
Patients demographics				
**Gender, no. (%) (missing=0)**				<0.001
Men	195 (59.3%)	165 (62.7%)	17 (25.8%)	
Women	134 (40.7%)	98 (37.3%)	49 (74.2%)	
**Age, (Mean ± SD) (missing=0)**	62.65 ± 12	63.6 ± 11	58.7 ± 14.7	0.007
**Treating center (missing=0)**				<0.001
**B**	100 (30%)	66 (25%)	34 (52%)	
**E**	229 (70%)	197 (75%)	32 (48%)	
Primary tumor characteristics and treatment				
**Pathological T stage**				<0.001
T1-T2	66 (24%)	41 (16.7%)	25 (86.2%)	
T3-4	209 (76%)	205 (83.3%)	4 (13.8%)	
Missing	54	17	37	
**Lymph Node status of primary tumor**				<0.001
Positive lymph node	213 (65.3%)	185 (71.2%)	28 (42.4%)	
Negative lymph node	113 (34.7%)	75 (28.8%)	38 (57.6%)	
Missing	3	3	0	
**Neoadjuvant chemotherapy (missing=0)**				0.883
No	223 (67.8%)	179 (68.1%)	44 (66.7%)	
Yes	106 (32.2%)	84 (31.9%)	22 (33.3%)	
**Adjuvant chemotherapy (missing=0)**				0.258
No	126 (36.7%)	105 (39.9%)	21 (31.8%)	
Yes	218 (66.3%)	158 (60.1%)	60 (68.2%)	
Liver metastases characteristics and treatment				
**Disease-free interval (missing=0)**				<0.001
≤12 months,	116 (35.3%)	74 (28.1%)	42 (63.6%)	
>12 months	213 (64.7%)	189 (71.9%)	24 (36.4%)	
**Number of LM Median [IQR] (missing=0)**	2 [3]	2 [3]	1 [2]	0.107
**Number of LM: >1 liver metastasis, no. (%) (missing=0)**				0.0096
Uninodular	118 (35.9%)	85 (32.3%)	33 (50%)	
Multinodular	211 (64.1%)	178 (67.7%)	33 (50%)	
**Size of LM: Median [IQR] (mm) (missing=0)**	29 [20]	27.5 [20]	31.5 [20]	0.360
**Size of LM, no. (%) (missing=0)**				0525
Largest <50 mm	247 (75.1%)	195 (74.1%)	52 (78.8%)	
Largest ≥50 mm	82 (24.9%)	68 (25.9%%)	14 (21.2%)	
**Preoperative chemotherapy (missing=0)**				0.499
No	69 (21%)	53 (20.2%)	16 (24.2%)	
Yes	260 (79%)	210 (79.8%)	50 (75.8%)	
**Extent of hepatectomy (missing=0)**				0.012
Minor (<3 segments)				
Major (≥3 segments)	142 (43.2%)	123 (46.8%)	19 (29.2%)	
**Postoperative chemotherapy (missing=0)**				<0.001
No	241 (73.3%)	211 (80.2%)	30 (45.5%)	
Yes	88 (26.7%)	52 (19.8%)	36 (54.5%)	
Histology of liver metastases				
Resection margins				0.181
R0 (≥1mm)	252 (77.3%)	199 (75.7%)	53 (84.1%)	
R1 (<1 mm)	74 (22.7%)	64 (24.3%)	10 (15.9%)	
Missing	3	0	3	
**Histological growth pattern (missing=0)**				0.470
**dHGP**	58 (18%)	49 (18.6%)	9 (13.6%)	
**Non-dHGP**	271 (82%)	214 (81.4%)	57 (86.4%)	

P-values are from Fisher’s exact tests. Counts are reported along with their percentages between brackets. Missing data are indicated.

CRLM, colorectal liver metastases; dHGP, desmoplastic histopathological growth pattern; LM, liver metastases; NCRLM, non-colorectal liver metastases; IQR, interquartile range; SD, standard deviation.

**Figure 2:**
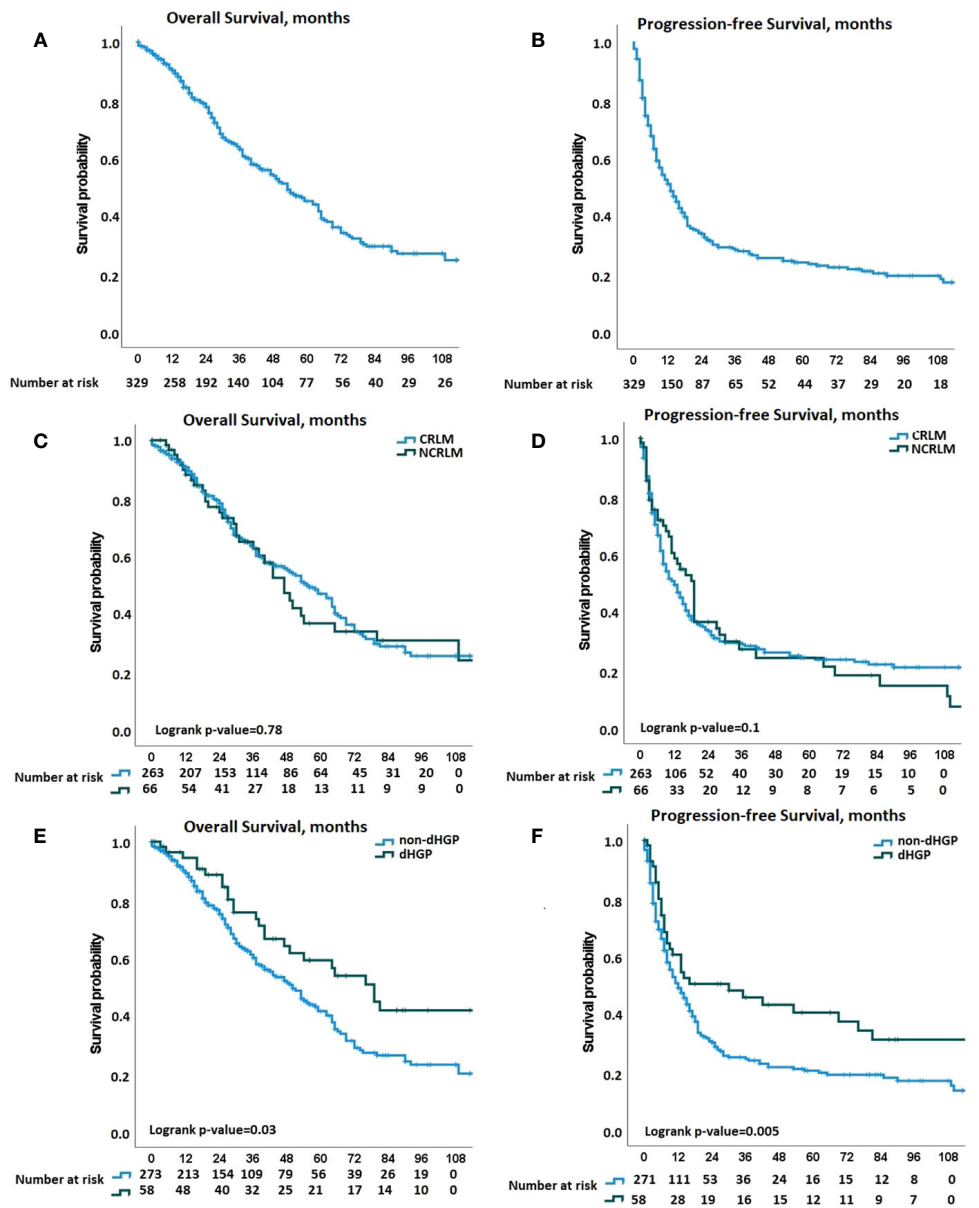
Kaplan-Meier curves for overall survival (OS) and progression-free survival (PFS) comparisons. **(A)** Kaplan-Meier curves displaying the overall survival (OS) probability in the whole population. **(B)** Kaplan-Meier curves displaying the progression-free survival (PFS) probability in the whole population. **(C, D)** Kaplan-Meier curves displaying the OS and PFS, respectively, probabilities according to the origin of liver metastases [Colorectal liver metastases CRLM vs non-CRLM (NCRLM)]. **(E, F)** Kaplan-Meier curves displaying the OS and PFS, respectively, probabilities according to histopathological growth pattern (HGP) group, (desmoplastic HGP (dHGP) vs non-dHGP).

**Figure 3:**
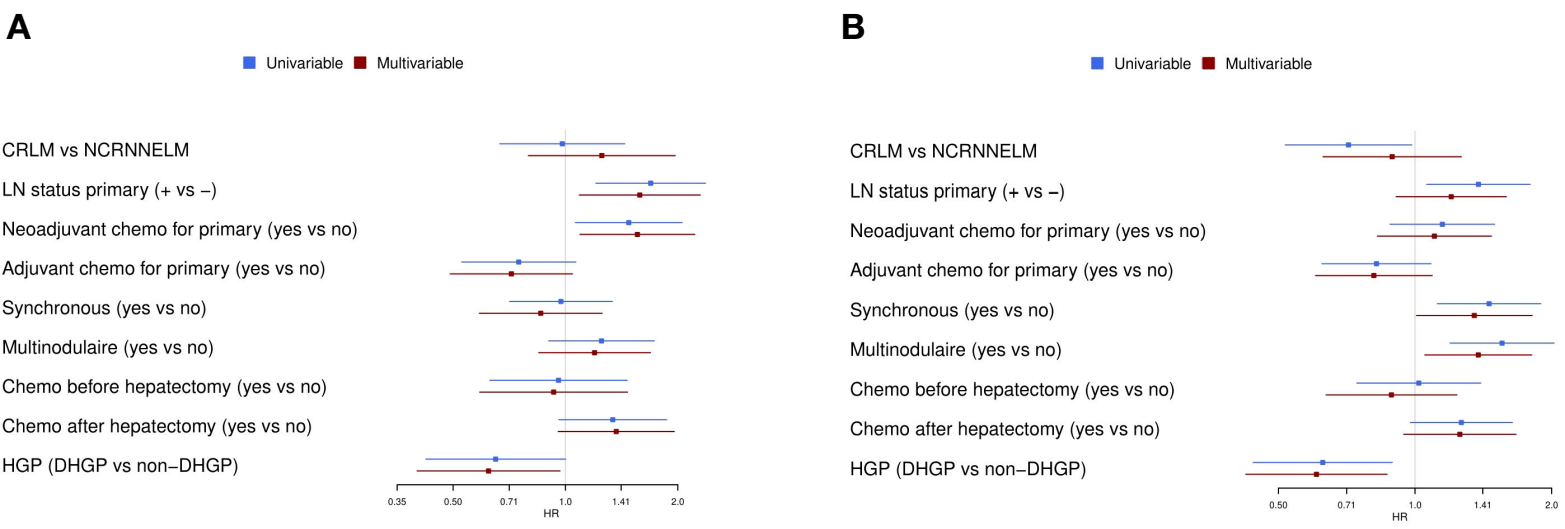
Univariable and multivariable analyses. **(A)** Univariable and multivariable Cox regression analyses for OS. **(B)** Univariable and multivariable Cox regression analyses for PFS. Chemo, chemotherapy; CI, confidence interval; CRC: colorectal cancer; HGP, histopathological growth pattern (dHGP: desmoplastic HGP); H&E, hematoxylin/eosin; HR, Hazard Ratio; LM, liver metastases; LN, lymph node; OS, overall survival; PFS, progression-free survival.

The median follow-up was 5.5 years for OS and 6.9 years for PFS. In the overall population, 3- and 5-year OS were 63.1% and 44.5%, respectively, and 3- and 5-year PFS were 21.5% and 15.5%, respectively (**Figures 2A, B**). No significant differences were observed for postoperative outcomes between patients who underwent surgery for colorectal versus non-colorectal LM. Three-year and 5-year OS were 63.3% and 46.8%, respectively, in patients with colorectal LM, and 62.5% and 35.2%, respectively, in patients with non-colorectal LM (p=0.78) (**Figure 2C**), and 3- and 5-year PFS were 20.0% and 14.2%, respectively, in patients with colorectal LM, and 27.1% and 20.3%, respectively, in patients with non-colorectal LM (p=0.1) (**Figure 2D**). In the entire cohort of patients with colorectal and non-colorectal LM, significantly better postoperative survival was observed in patients who underwent surgery for dHGP LM as compared to non-dHGP. Three- and 5-year OS were 74.0% and 57.3%, respectively, in patients with dHGP LM, as compared to 60.6% and 41.3%, respectively, in patients with non-dHGP (p=0.03) (**Figure 2E**), and 3- and 5-year PFS were 36.8% and 32.2%, respectively, in patients with dHGP LM, as compared to 18.3% and 11.8%, respectively, in patients with non-dHGP LM (p=0.005) (**Figure 2F**). When compared with all of the traditional risk factors in patients with LM and irrespective of the primary cancer origin, HGP was found to be the most significant prognostic factor for postoperative survival, in univariate and multivariate analyses (**Figure 3**; **Supplementary Tables 3, 4**). In multivariable analysis, dHGP was positively associated with postoperative OS (adjusted HR: 0.62; 95% CI: [0.40-0.97]; p=0.035) and PFS (adjusted HR: 0.61; 95% CI: [0.42-0.87], p=.006) (**Figure 3**).

Of note, KRAS mutational status represented another prognostic factor in patients who underwent surgery for colorectal LM. Among the 200 patients in whom this analysis was available, there were 116 patients (58%) with KRAS wild-type status and 84 (42%) with KRAS mutated status. The median OS in the mutated KRAS group was 30 months (95% CI: 19- 44) as compared to 59 months (95% CI: 34-84) in the wild-type group, with 3-year and 5-year OS of 45.4% and 33.4%, respectively, in the KRAS mutated group as compared with 65% and 46.6%, respectively, in the wild-type group (p=0.161). The median PFS in the mutated KRAS group was 8 months (95% CI: 5-11) vs 9 months (95% CI: 5-13) in the wild-type group, with 3-year and 5-year PFS of 21.7% and 19.9%, respectively, in the mutated KRAS group as compared to 29.2% and 27.8%, respectively, in the wild-type group (p=0.228).

Overall, among the patients with postoperative recurrences, we observed liver-only recurrences in 15 of the 35 patients with dHGP (43%) and in 91 of the 198 patients with non-dHGP (46%). A trend toward a higher rate of R1 resection was observed in the non-dHGP cases. In the entire population, including colorectal and non-colorectal LM, R1 resections were observed in 7 of the 49 patients with dHGP (14.3%) and in 57 of the 214 patients with non-dHGP (26.6%) (p=0.095).

## Discussion

The results of surgery for LM of colorectal or non-colorectal origin remains difficult to predict. This is illustrated in the present series by the heterogeneous postoperative outcomes, with 35% to 45% of patients experiencing prolonged survival and, in contrast, approximately 50% of patients with disease recurrence within one year. Globally, we observed relatively similar postoperative outcomes in patients who underwent resection of colorectal or non-colorectal LM. Clearly, this observation should be interpreted with caution, considering the major differences in tumor biology and metastatic behavior in these various tumor. In addition, this observation is most probably biased in this series, due the overrepresentation of colorectal LM and the overrepresentation of breast LM in the non-colorectal group. As expected also, the surgical selection has been much more stringent in patients with non-colorectal LM, as illustrated by less advanced primary tumors and metastatic stages, more frequent use of systemic treatment, and longer disease-free intervals in these patients as compared with those with colorectal LM. However, beyond these biases, this finding suggests that, in different primary cancers, the development of isolated LM can either reflect a true oligometastatic disease or, in contrast, correspond to the first manifestation of a diffuse metastatic spread in which occult disease will be responsible for relapse. In this context, the possibility of distinguishing between these metastatic behavior patterns would represent major progress toward the identification of patients who will benefit from LM-targeted therapies as opposed to those who will require a systemic treatment approach.

We confirmed the poor accuracy of traditional predictive and prognostic factors and scores in patients who underwent surgical resection of colorectal or non-colorectal LM (3, 27–30). This indicates that classical markers, essentially relying on cancer stage and aggressiveness, such as the primary tumor stage, the mutational status, the kinetics and the extent of LM, are not sufficient to depict the overall tumor biology and to predict the type of metastatic progression. It is indeed expected that, in each individual case, metastatic aggressiveness not only relies on intrinsic tumor characteristics but also on multiple interactions between cancer cells, host responses, and microenvironment. In this setting, the HGP of LM represents an attractive candidate marker, as it potentially integrates information on all of these complex biological interactions.

Importantly, distinct HGPs of LM have now been clearly described in consensus guidelines (26), allowing for reproducible identification and scoring. Furthermore, in all series of patients undergoing surgery for LM of various non-neuroendocrine origins, a similar prognostic impact of HGP has been reported, with significantly better outcomes in patients who undergo surgery for dHGP LM as compared to patients with non-dHGP or rHGP LM (25, 31).

To evaluate the potential value of HGP of LM as a cancer-agnostic marker, we regrouped patients who underwent surgery for colorectal or non-colorectal LM. In this series, we first confirmed that HGP was scorable in all of these cases. Overall, similar ratios of dHGP and non-dHGP were found in LM of colorectal or non-colorectal origin, with the exception of the LM of breast cancer origin in which a higher rate of rHGP was observed (24). This indicates that, besides the host responses and the specifics of the liver microenvironment, the characteristics of the primary tumor retain a role in the interplay leading to the development of LM with different HGPs.

Regardless of whether the cancer was of colorectal or non-colorectal origin, we found that HGP of LM was an independent prognostic factor for postoperative survival and progression, representing the most robust factor associated with OS and PFS as compared to all classical variables. Moreover, we observed that LM HGP had a similar extent of prognostic impact in colorectal and non-colorectal cases, supporting the hypothesis that, when LM are established, their microarchitecture accurately reflects the mode of metastatic progression with which they are associated. As a surrogate marker of the overall metastatic aggressiveness, HGP could also indicate the local aggressiveness of LM. We observed similar rates of liver-only recurrence in the dHGP and non-dHGP groups, but a trend toward a higher rate of R1 resections in the non-dHGP group (32, 33).

Taken together, these data highlight the value of HGP as a promising biomarker in patients who are candidates for surgery for LM, to improve the individualization of therapeutic decision making and to better understand the mechanisms associated with different types of metastatic progression. Currently, HGP status cannot be used for individual decision making, as it is only assessable by pathological examination of the surgical samples, requiring the analysis of the entire TLI. Progress has been made recently to make this information available prior to surgery, including the development of multi-parameter predictive models for HGP (34) and of new dedicated imaging algorithms (35–37). One step further, the in-depth analysis of the cellular and molecular components of the distinct HGPs could provide new insights into the mechanisms implicated in their development and in how these mechanisms reflect overall metastatic behavior. At the present, however, it has been proposed that dHGP, characterized by a peritumoral fibrous reaction and abundant immune infiltrate, and considered to be a host-driven pattern (38), preferentially reflects an oligometastatic or argometastatic progression, while rHGP, characterized by an infiltrating profile with poor or absent immune reaction, could reflect a more aggressive and diffusely metastatic progression. In this phenomenon, anti-tumor immune reaction is expected to play a central role. Although the nature and extent of immune infiltrates present in dHGP LM still remain incompletely documented (39), these cells could be critical to generating an anti-tumor reaction to control metastatic progression, both at the local and systemic levels (40, 41). Accordingly, the qualification and quantification of these immune cells could contribute to a better understanding of the mechanisms of tumor progression and, potentially, to identification of new therapeutic targets. It remains true that there is significant overlap regarding the postoperative prognosis when comparing patients with dHGP with those with non-dHGP, suggesting that HGP essentially represents a surrogate marker of metastatic biology. Therefore, to elucidate the role of immune infiltrates, it is necessary to better qualify their nature. For instance, this could be analyzed in patients with dHGP LM by comparing the quality of peritumor immune cells in patients with a prolonged postoperative survival and in those with a rapid relapse.

In conclusion, the tumor microenvironment of LM, reflected by HGP, appears to be a reliable surrogate marker of metastatic behavior in patients with isolated LM from different primary cancer origins. Therefore, prediction of HGP with non-invasive methods represents an important step to provide a better individualization of therapeutic decision making in these patients. The fact that the prognostic impact of HGP was verified independently of the type of primary tumor suggests that this parameter may reflect central mechanisms related to tumor immunogenicity and host-tumor interactions, offering new opportunities for future research.

## Data availability statement

The raw data supporting the conclusions of this article will be made available by the authors, without undue reservation.

## Author contributions

AB: Conceptualization, Data curation, Formal Analysis, Writing – original draft, Writing – review & editing. FR: Formal Analysis, Writing – original draft, Writing – review & editing, Validation. VL: Writing – review & editing, Data curation, Investigation, Methodology. AE: Data curation, Writing – review & editing. PD: Data curation, Writing – review & editing, Formal Analysis. LC: Data curation, Formal Analysis, Writing – review & editing. DL: Data curation, Writing – review & editing. AH: Data curation, Writing – review & editing, Formal Analysis. JV: Writing – review & editing, Methodology, Supervision, Validation. LD: Methodology, Supervision, Validation, Writing – review & editing, Investigation. CD: Writing – review & editing, Conceptualization, Data curation, Formal Analysis, Writing – original draft. PV: Conceptualization, Writing – original draft, Writing – review & editing, Investigation, Methodology. VD: Conceptualization, Writing – original draft, Writing – review & editing, Data curation, Formal Analysis.

## References

[1] Van Cutsem E Cervantes A Adam R Sobrero A Van Krieken JH Aderka D. ESMO consensus guidelines for the management of patients with metastatic colorectal cancer. Ann Oncol (2016) 27:1386–422. doi: 10.1093/annonc/mdw235 27380959

[2] LiverMetSurvey ARCAD. Available at: https://livermetsurvey-arcad.org/ (Accessed May 8, 2019).

[3] Bohlok A Lucidi V Bouazza F Daher A Germanova D Van Laethem JL. The lack of selection criteria for surgery in patients with non-colorectal non-neuroendocrine liver metastases. World J Surg Oncol (2020) 18. doi: 10.1186/S12957-020-01883-Y PMC7249425 32450872

[4] Imamura H Seyama Y Kokudo N Maema A Sugawara Y Sano K. One thousand fifty-six hepatectomies without mortality in 8 years. Arch Surg (2003) 138:1198–206. doi: 10.1001/ARCHSURG.138.11.1198 14609867

[5] Lucidi V Bohlok A Liberale G Bez M Germanova D Bouazza F. Extended time interval between diagnosis and surgery does not improve the outcome in patients operated for resection or ablation of breast cancer liver metastases. Eur J Surg Oncol (2020) 46:229–34. doi: 10.1016/J.EJSO.2019.10.016 31677938

[6] Adam R Chiche L Aloia T Elias D Salmon R Rivoire M. Hepatic resection for noncolorectal nonendocrine liver metastases: analysis of 1,452 patients and development of a prognostic model. Ann Surg (2006) 244:524–35. doi: 10.1097/01.sla.0000239036.46827.5f PMC1856551 16998361

[7] Bauschke A Altendorf-Hofmann A Homman M Manger T Pertschy J Helfritzsch H. Surgical treatment of liver metastases from non-colorectal non-neuroendocrine carcinomas. J Cancer Res Clin Oncol (2022) 148:503–15. doi: 10.1007/s00432-021-03631-5 PMC8800927 33880657

[8] Yedibela S Gohl J Graz V Pfaffenberger MK Merkel S Hohenberger W. Changes in indication and results after resection of hepatic metastases from noncolorectal primary tumors: A single-institutional review. Ann Surg Oncol (2005) 12:778–85. doi: 10.1245/ASO.2005.11.018 16132374

[9] Hoffmann K Bulut S Tekbas A Hinz U Büchler MW Schemmer P. Is hepatic resection for non-colorectal, non-neuroendocrine liver metastases justified? Ann Surg Oncol (2015) 22 Suppl 3:S1083–92. doi: 10.1245/s10434-015-4775-x 26242369

[10] Hellman S Weichselbaum RR. Oligometastases. J Clin Oncol (1995) 13:8–10. doi: 10.1200/JCO.1995.13.1.8 7799047

[11] Weichselbaum RR Hellman S. Oligometastases revisited. Nat Rev Clin Oncol (2011) 8:378–82. doi: 10.1038/nrclinonc.2011.44 21423255

[12] Szturz P Vermorken JB. Steering decision making by terminology: oligometastatic versus argometastatic. Br J Cancer (2022) 127:587–91. doi: 10.1038/S41416-022-01879-3 PMC9381792 35715637

[13] Viganò L Capussotti L Lapointe R Barroso E Hubert C Giuliante F. Early recurrence after liver resection for colorectal metastases: Risk factors, prognosis, and treatment. A LiverMetSurvey-based study of 6,025 patients. . Ann Surg Oncol (2014) 21:1276–86. doi: 10.1245/s10434-013-3421-8 24346766

[14] Viganò L Gentile D Galvanin J Corleone P Costa G Cimino M. Very early recurrence after liver resection for colorectal metastases: incidence, risk factors, and prognostic impact. J Gastrointest Surg (2022) 26:570–82. doi: 10.1007/S11605-021-05123-W 34508293

[15] Ecker BL Lee J Saadat LV Aparicio T Buisman FE Balachandran VP. Recurrence-free survival versus overall survival as a primary endpoint for studies of resected colorectal liver metastasis: a retrospective study and meta-analysis. Lancet Oncol (2022) 10:1332–1342. doi: 10.1016/s1470-2045(22)00506-x 36058227

[16] Malik HZ Gomez D Wong V Al-Mukthar A Toogood GJ Lodge JPA. Predictors of early disease recurrence following hepatic resection for colorectal cancer metastasis. Eur J Surg Oncol (2007) 33:1003–9. doi: 10.1016/j.ejso.2007.01.005 17350218

[17] Takahashi S Konishi M Kinoshita T Gotohda N Kato Y Saito N. Predictors for early recurrence after hepatectomy for initially unresectable colorectal liver metastasis. J Gastrointest Surg (2013) 17:939–48. doi: 10.1007/S11605-013-2162-0/FIGURES/2 23400510

[18] Vermeulen PB Colpaert C Salgado R Royers R Hellemans H Van den Heuvel E. Liver metastases from colorectal adenocarcinomas grow in three patterns with different angiogenesis and desmoplasia. J Pathol (2001) 195:336–42. doi: 10.1002/path.966 11673831

[19] Galjart B Nierop PMH van der Stok EP van den Braak RRJC Höppener DJ Daelemans S. Angiogenic desmoplastic histopathological growth pattern as a prognostic marker of good outcome in patients with colorectal liver metastases. Angiogenesis (2019) 22:355–68. doi: 10.1007/s10456-019-09661-5 PMC6475515 30637550

[20] Höppener DJ Nierop PMH Herpel E Rahbari NN Doukas M Vermeulen PB. Histopathological growth patterns of colorectal liver metastasis exhibit little heterogeneity and can be determined with a high diagnostic accuracy. Clin Exp Metastasis (2019) 36:311–9. doi: 10.1007/s10585-019-09975-0 PMC6611753 31134394

[21] Höppener DJ Galjart B Nierop PMH Buisman FE van der Stok EP van den Braak RRJC. Histopathological growth patterns and survival after resection of colorectal liver metastasis: an external validation study. JNCI Cancer Spectr (2021) 5. doi: 10.1093/JNCICS/PKAB026 PMC8152695 34056541

[22] Barnhill R Vermeulen P Daelemans S van Dam P-J Roman-Roman S Servois V. Replacement and desmoplastic histopathological growth patterns: A pilot study of prediction of outcome in patients with uveal melanoma liver metastases. J Pathol Clin Res (2018) 4:227–40. doi: 10.1002/cjp2.105 PMC6174621 29917326

[23] Barnhill R Dam P Vermeulen P Champenois G Nicolas A Rawson RV. Replacement and desmoplastic histopathological growth patterns in cutaneous melanoma liver metastases: frequency, characteristics, and robust prognostic value. J Pathol Clin Res (2020) 6:195–206. doi: 10.1002/cjp2.161 32304183 PMC7339161

[24] Bohlok A Vermeulen P Leduc S Latacz E Botzenhart L Richard F. Association between the histopathological growth patterns of liver metastases and survival after hepatic surgery in breast cancer patients. NPJ Breast Cancer (2020) 6. doi: 10.1038/S41523-020-00209-1 PMC7749172 33339824

[25] Meyer Y Bohlok A Höppener D Galjart B Doukas M Grünhagen DJ. Histopathological growth patterns of resected non-colorectal, non-neuroendocrine liver metastases: a retrospective multicenter studyss. Clin Exp Metastasis (2022) 39:433–42. doi: 10.1007/S10585-022-10153-Y 35124739

[26] Latacz E Höppener D Bohlok A Leduc S Tabariès S Fernández Moro C. Histopathological growth patterns of liver metastasis: updated consensus guidelines for pattern scoring, perspectives and recent mechanistic insights. Br J Cancer (2022) 127(6):988–1013. doi: 10.1038/s41416-022-01859-7 35650276 PMC9470557

[27] Duran Derijckere I Levillain H Bohlok A Mathey C Nezri J Muteganya R. The metabolic clinical risk score as a new prognostic model for surgical decision-making in patients with colorectal liver metastases. J Surg Oncol (2020) 121:350–6. doi: 10.1002/jso.25763 31721228

[28] Ayez N Lalmahomed ZS van der Pool AEM Vergouwe Y van Montfort K de Jonge J. Is the clinical risk score for patients with colorectal liver metastases still useable in the era of effective neoadjuvant chemotherapy? Ann Surg Oncol (2011) 18:2757–63. doi: 10.1245/s10434-011-1819-8 PMC3171666 21638093

[29] Maithel SK Gönen M Ito H Dematteo RP Allen PJ Fong Y. Improving the clinical risk score: an analysis of molecular biomarkers in the era of modern chemotherapy for resectable hepatic colorectal cancer metastases. Surgery (2012) 151:162–70. doi: 10.1016/J.SURG.2011.07.020 21982065

[30] Takeda Y Mise Y Matsumura M Hasegawa K Yoshimoto J Imamura H. Accuracy of modern clinical risk score including RAS status changes based on whether patients received perioperative chemotherapy for colorectal liver metastases. World J Surg (2021) 45:2176–84. doi: 10.1007/S00268-021-05976-X/FIGURES/3 33880608

[31] Meyer Y Bohlok A Olthof P Donckier V Doukas M Lucidi V. Histopathological growth patterns of neuroendocrine tumor liver metastases. Clin Exp Metastasis (2023) 40:227–34. doi: 10.1007/S10585-023-10211-Z PMC10232551 37183203

[32] Nierop PMH Höppener DJ van der Stok EP Galjart B Buisman FE Balachandran VP. Histopathological growth patterns and positive margins after resection of colorectal liver metastases. HPB (2020) 22:911–9. doi: 10.1016/j.hpb.2019.10.015 PMC7888172 31735649

[33] Bohlok A Inchiostro L Lucidi V Vankerckhove S Hendlisz A Van Laethem JL. Tumor biology reflected by histological growth pattern is more important than surgical margin for the prognosis of patients undergoing resection of colorectal liver metastases. Eur J Surg Oncol (2022) 49:217–24. doi: 10.1016/J.EJSO.2022.08.006 36031469

[34] Höppener DJ Stook JLPL Galjart B Nierop PMH Nagtegaal ID Vermeulen PB. The relationship between primary colorectal cancer histology and the histopathological growth patterns of corresponding liver metastases. BMC Cancer (2022) 22:911. doi: 10.1186/S12885-022-09994-3 35996090 PMC9394040

[35] Cheng J Wei J Tong T Sheng W Zhang Y Han Y. Prediction of histopathologic growth patterns of colorectal liver metastases with a noninvasive imaging method. Ann Surg Oncol (2019) 26:4587–98. doi: 10.1245/S10434-019-07910-X 31605342

[36] Wei S Han Y Zeng H Ye S Cheng J Chai F. Radiomics diagnosed histopathological growth pattern in prediction of response and 1-year progression free survival for colorectal liver metastases patients treated with bevacizumab containing chemotherapy. Eur J Radiol (2021) 142. doi: 10.1016/J.EJRAD.2021.109863 34343846

[37] Latacz E van Dam PJ Vanhove C Llado L Descamps B Ruiz N. Can medical imaging identify the histopathological growth patterns of liver metastases? Semin Cancer Biol (2020) 142. doi: 10.1016/j.semcancer.2020.07.002 32735852

[38] Moro CF Harrizi S Hamidi Y Geyer N Kuznyecov D Tidholm-Qvist E. An idiosyncratic, zonated stroma encapsulates desmoplastic liver metastases and originates from injured liver. medRxiv (2022) 14. doi: 10.1101/2022.08.24.22279162 PMC10439160 37596278

[39] Messaoudi N Henault D Stephen D Cousineau I Simoneau E Rong Z. Prognostic implications of adaptive immune features in MMR-proficient colorectal liver metastases classified by histopathological growth patterns. Br J Cancer (2022) 126:1329–38. doi: 10.1038/S41416-021-01667-5 PMC9043179 34980880

[40] Mlecnik B Van Den Eynde M Bindea G Church SE Vasaturo A Fredriksen T. Comprehensive intrametastatic immune quantification and major impact of immunoscore on survival. JNCI J Natl Cancer Inst (2018) 110:97–108. doi: 10.1093/JNCI/DJX123 28922789

[41] Yu J Green MD Li S Sun Y Journey SN Choi JE. Liver metastasis restrains immunotherapy efficacy viamacrophage-mediated T cell elimination. Nat Med (2021) 27:152. doi: 10.1038/S41591-020-1131-X 33398162 PMC8095049

