# Supplementary material for: Histopathological growth pattern of liver metastases as an independent marker of metastatic behavior in different primary cancers
Source: Front Oncol. 2023 Oct 27;13:1260880. doi: 10.3389/fonc.2023.1260880 (PMC10641477; doi:10.3389/fonc.2023.1260880)
Supplement: Supplementary file 1 [file DataSheet_1.pdf]

**SUPPLEMENTARY MATERIAL FOR**

**Bohlok A, et al. Histopathological growth pattern as a cancer-agnostic prognostic marker in liver metastases**

**A. Supplementary methods___________________________________________p2**

**B. Supplementary tables_____________________________________________p4**

**C. Supplementary figure_____________________________________________p8**

## A. Supplementary methods:

Definitions of histological growth pattern:

For each patient, all of the available hematoxylin/eosin (HE)-stained sections of the tumor-to-liver interface (TLI) in all available LM were examined using light microscopy, blinded to clinical data. Desmoplastic HGP (dHGP) was defined as the presence of a peritumoral fibrotic rim, with no direct cancer cells-to-liver cells interactions. The fibrous rim frequently contained a dense lymphocytic infiltrate and proliferative bile ducts. LM with a dHGP are vascularized by a process of angiogenesis. Replacement HGP (rHGP) was defined as the absence of a peritumoral fibrous rim, in which cancer cells are in contact with the hepatocytes, coopting the liver sinusoidal vasculature for blood supply. In this pattern, there is often only a minimal or even absent inflammatory infiltrate. A third pattern, defined as the pushing HGP (pHGP), was rarely observed. In these cases, the LM pushes away the surrounding liver tissue, without growth in the hepatic parenchyma and without peritumoral fibrotic reaction. LM were scored as dHGP when this pattern represented 100% of the TLI of all resected LM and as non-dHGP when any percentage of rHGP or pHGP was present.

Statistical analysis:

Associations between HGP or origin of the primary tumor and clinicopathologic characteristics were assessed with Fisher's exact tests. Progression-free survival (PFS) was defined as the time between surgery for LM, and the last follow-up, or the first recurrence or the death from any cause. Overall survival (OS) was defined as the time between surgery for LM and the last follow-up or death from any cause. Time-to-event analyses were performed using stratified log-rank test and Cox proportional hazard regressions. The proportional hazard assumptions were checked by analyzing the Schoenfeld residuals through the cox.zph function from the R survival package. Potential confounder factors were inventoried based on a literature search and selected in the multivariable analysis whenever associated with the outcome at the univariable level or for biological relevance. Median follow-up was

computed using the reverse Kaplan-Meier estimator and was curtailed at 9 years given the low number of patients after that timepoint. All analyses were performed using R 4.0.2. P-values were 2-sided and considered significant when $p<.05$.

## B. Supplementary Tables:

**Supplementary Table 1: Primary tumor origins among non-colorectal liver metastases included in the study**

| **Primary cancer origins of non-colorectal liver metastases** | |
|---|---|
| Anal carcinoma | 1 (1.6%) |
| Breast | 36 (54.5%) |
| Ear, nose, and throat | 3 (4.5%) |
| Esophagus | 2 (3.0%) |
| Gastrointestinal stromal tumor | 6 (9.1%) |
| Hemangiopericytoma | 1 (1.6%) |
| Kidney | 1 (1.6%) |
| Leiomyoma | 2 (3.0%) |
| Melanoma | 3 (4.5%) |
| Ovary | 3 (4.5%) |
| Pancreas | 4 (6.1%) |
| Small bowel | 2 (3.0%) |
| Testicle | 2 (3.0%) |

**<u>Supplementary Table 2</u>: Clinicopathologic characteristics among patients with desmoplastic and non-desmoplastic HGP liver metastases**

| Characteristics | Non-dHGP (n=271; 82%) | dHGP (n=58; 18%) | p |
|---|---|---|---|
| **Gender, no. (%) (missing=0)** | | | 0.663 |
| Men | 148 (54.6) | 34 (58.6%) | |
| Women | 123 (45.4) | 24 (41.4) | |
| **Pathological T stage** | | | 0.098 |
| T1-T2 | 59 (26%) | 7 (14.6%) | |
| T3-4 | 168 (74%) | 41 (85.4%) | |
| Missing=54 | 44 | 10 | |
| **Lymph node status of primary tumor no. (%)** | | | 0.446 |
| Positive lymph node | 96 (35.7%) | 17 (29.8%) | |
| Negative lymph node | 173 (64.3%) | 40 (70.2%) | |
| Missing | 2 | 1 | |
| **Disease-free interval no. (%) (missing=0)** | | | 0.364 |
| ≤12 months, | 172 (63.5%) | 41 (70.7%) | |
| >12 months | 99 (36.5%) | 17 (29.3%) | |
| **Number of CRLM: >1 liver metastasis, no. (%) (missing=0)** | | | 0.764 |
| Uninodular | 96 (35.4%) | 22 (37.9%) | |
| Multinodular | 175 (64.6%) | 36 (62.1%) | |
| **Size of CRLM, no. (%) (missing=0)** | | | 1 |
| Largest <50 mm | 203 (74.9%) | 44 (75.9%) | |
| Largest ≥50 mm | 68 (25.1%) | 14 (24.1%) | |
| **Preoperative chemotherapy (missing=0)** | | | 0.158 |
| No | 61 (22.5%) | 8 (13.8%) | |
| Yes | 210 (77.5%) | 50 (86.2%) | |
| **Primary tumor origin** | | | 0.002 |

| | | | |
|---|---|---|---|
| Anal carcinoma | 0 (0.0) | 1 (1.7) | |
| Breast | 35 (12.9) | 1 (1.7) | |
| Colorectal | 214 (79.0) | 49 (84.5) | |
| Ear, nose, and throat | 3 (1.1) | 0 (0.0) | |
| Esophagus | 2 (0.7) | 0 (0.0) | |
| Gastrointestinal stromal tumor | 3 (1.1) | 3 (5.2) | |
| Hemangiopericytoma | 1 (0.4) | 0 (0.0) | |
| Kidney | 0 (0.0) | 1 (1.7) | |
| Leomyoma | 2 (0.7) | 0 (0.0) | |
| Melanoma | 3 (1.1) | 0 (0.0) | |
| Ovary | 2 (0.7) | 1 (1.7) | |
| Pancreas | 4 (1.5) | 0 (0.0) | |
| Small bowel | 2 (0.7) | 0 (0.0) | |
| Testicles | 0 (0.0) | 2 (3.4) | |

**Supplementary Table 3: Univariable and multivariable analysis of factors affecting overall survival**

| **Overall survival analysis** | **Univariable** | | | | **Multivariable** | | | |
|---|---|---|---|---|---|---|---|---|
| **Variables** | **events** | **samples** | **HR (95% CI)** | **p** | **events** | **samples** | **HR (95% CI)** | **p** |
| CRC vs non-CRC | 162 | 329 | 0.98 (0.67-1.44) | 0.922 | 161 | 326 | 1.25 (0.80-1.97) | 0.332 |
| LN status primary cancer (+ vs -) | 161 | 326 | 1.69 (1.21-2.37) | 0.002 | 161 | 326 | 1.58 (1.09-2.30) | 0.016 |
| Neoadjuvant chemo for primary (yes vs no) | 162 | 329 | 1.48 (1.06-2.05) | 0.020 | 161 | 326 | 1.56 (1.09-2.22) | 0.014 |
| Adjuvant chemo for primary (yes vs no) | 162 | 329 | 0.75 (0.53-1.07) | 0.109 | 161 | 326 | 0.72 (0.49-1.05) | 0.084 |
| Synchronous (yes vs no) | 162 | 329 | 0.97 (0.71-1.34) | 0.864 | 161 | 326 | 0.86 (0.59-1.25) | 0.431 |
| Multinodular (yes vs no) | 162 | 329 | 1.25 (0.90-1.73) | 0.181 | 161 | 326 | 1.20 (0.85-1.69) | 0.307 |
| Chemo before hepatectomy (yes vs no) | 162 | 329 | 0.96 (0.63-1.46) | 0.844 | 161 | 326 | 0.93 (0.59-1.47) | 0.754 |
| Chemo after hepatectomy (yes vs no) | 162 | 329 | 1.34 (0.96-1.87) | 0.086 | 161 | 326 | 1.37 (0.96-1.96) | 0.086 |
| HGP (dHGP vs non−dHGP) | 162 | 329 | 0.65 (0.42-1.00) | 0.050 | 161 | 326 | 0.62 (0.40-0.97) | 0.035 |

**Supplementary Table 4: Univariable and multivariable analysis of factors affecting progression-free survival**

| Progression-free survival analysis | Univariable | | | | Multivariable | | | |
|---|---|---|---|---|---|---|---|---|
| **Variables** | **events** | **samples** | **HR (95% CI)** | **p** | **events** | **samples** | **HR (95% CI)** | **p** |
| CRC vs non-CRC | 256 | 329 | 0.71 (0.52-0.98) | 0.039 | 254 | 326 | 0.89 (0.63-1.26) | 0.517 |
| LN status primary cancer (+ vs -) | 254 | 326 | 1.38 (1.06-1.79) | 0.016 | 254 | 326 | 1.20 (0.91-1.59) | 0.199 |
| Neoadjuvant chemo for primary (yes vs no) | 256 | 329 | 1.15 (0.88-1.50) | 0.307 | 254 | 326 | 1.10 (0.83-1.47) | 0.507 |
| Adjuvant chemo for primary (yes vs no) | 256 | 329 | 0.82 (0.62-1.09) | 0.167 | 254 | 326 | 0.81 (0.60-1.09) | 0.167 |
| Synchronous (yes vs no) | 256 | 329 | 1.46 (1.12-1.89) | 0.005 | 254 | 326 | 1.35 (1.01-1.81) | 0.045 |
| Multinodular (yes vs no) | 256 | 329 | 1.56 (1.19-2.03) | 0.001 | 254 | 326 | 1.38 (1.05-1.81) | 0.021 |
| Chemo before hepatectomy (yes vs no) | 256 | 329 | 1.02 (0.74-1.40) | 0.906 | 254 | 326 | 0.89 (0.64-1.24) | 0.480 |
| Chemo after hepatectomy (yes vs no) | 256 | 329 | 1.26 (0.98-1.64) | 0.075 | 254 | 326 | 1.26 (0.94-1.67) | 0.118 |
| HGP (dHGP vs non−dHGP) | 256 | 329 | 0.63 (0.44-0.89) | 0.009 | 254 | 326 | 0.61 (0.42-0.87) | 0.006 |

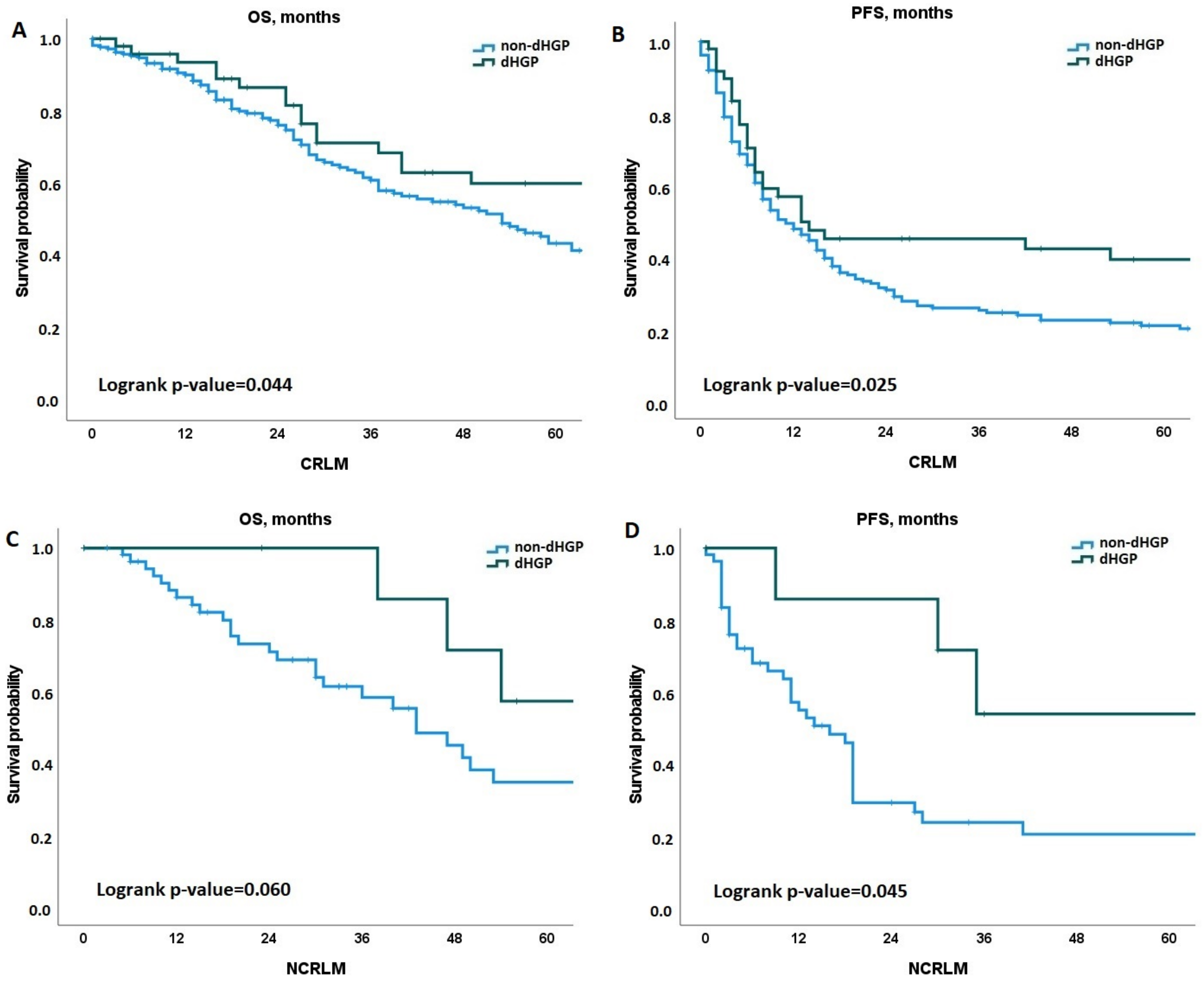


**Supplementary Figure 1:** Kaplan-Meier curves displaying the OS (A and C) and PFS (B and D) probabilities according to the histopathological growth pattern (dHGP vs non-dHGP) in patients with colorectal liver metastases (CRLM) (A and B) and in patients with non-CRLM (NCRLM) (C and D).
